# Durability of Nucleos(t)ide Analogues Treatment in Patients With Chronic Hepatitis B

**DOI:** 10.1097/MD.0000000000001341

**Published:** 2015-08-14

**Authors:** I-Cheng Lee, Cheuk-Kay Sun, Chien-Wei Su, Yuan-Jen Wang, Hung-Chuen Chang, Hui-Chun Huang, Kuei-Chuan Lee, Yi-Shin Huang, Chin-Lin Perng, Yuh-Hwa Liu, Chian-Sem Chua, Yu-Min Lin, Han-Chieh Lin, Yi-Hsiang Huang

**Affiliations:** From the Division of Gastroenterology, Department of Medicine, Taipei Veterans General Hospital (I-CL, C-WS, H-CH, K-CL, Y-SH, C-LP, H-CL, Y-HH); Faculty of Medicine, National Yang-Ming University School of Medicine (I-CL, C-WS, H-CH, K-CL); Division of Gastroenterology, Shin Kong Wu Ho-Su Memorial Hospital (C-KS, H-CC, Y-HL, C-SC, Y-ML); Health Care Center, Taipei Veterans General Hospital (Y-JW); and Institute of Clinical Medicine, National Yang-Ming University, Taipei, Taiwan (I-CL, C-WS, K-CL, Y-HH).

## Abstract

Long-term nucleos(t)ide analogues (NUCs) treatment is usually required for patients with chronic hepatitis B (CHB). However, whether discontinuation of NUCs is possible in selected patients remains debated. The aim of this study was to assess the durability of NUCs and predictors of sustained response after cessation of NUCs.

Ninety-three CHB patients (29 HBeAg-positive and 64 HBeAg-negative) from 2 medical centers in Taiwan with discontinuation of NUCs after a median of 3 years’ treatment were retrospectively reviewed. Fifteen (51.7%) HBeAg-positive and 57 (89.1%) HBeAg-negative patients achieved APASL treatment endpoints. Virological relapse (VR) and clinical relapse (CR) were defined according to APASL guidelines.

Achieving APASL endpoint was associated with longer median time to CR in HBeAg-positive patients, but not in HBeAg-negative cases. The cumulative 1-year VR and CR rates were 55.3% and 14.4% in HBeAg-positive patients, and 77.7% and 41.9% in HBeAg-negative patients, respectively. In HBeAg-negative patients, baseline HBV DNA >10^5^ IU/mL was the only predictor of VR (hazard ratio [HR] = 2.277, *P* = 0.019) and CR (HR = 3.378, *P* = 0.014). HBsAg >200 IU/mL at the end of treatment (EOT) was associated with CR (HR = 3.573, *P* = 0.023) in patients developing VR. HBeAg-negative patients with low baseline viral loads and low HBsAg levels at EOT had minimal risk of CR after achieving APASL treatment endpoint (*P* = 0.016).

The VR rate is high, but the risk of CR is low within 1 year with consolidation treatment after HBeAg seroconversion. Longer consolidation treatment to reduce the risk of VR should be considered in HBeAg-positive patients. As high risk of VR and CR, cessation of NUCs therapy could be considered only in selected HBeAg-negative patients.

## INTRODUCTION

Hepatitis B virus (HBV) infection is one of the most common infections in the world, with >350 million carriers worldwide.^1,2^ Patients with chronic hepatitis B (CHB), especially those with persistent HBV replication, may develop hepatic decompensation, cirrhosis, or hepatocellular carcinoma (HCC), leading to significant health impact.^3^ Currently nucleos(t)ide analogues (NUCs) are the mainstay of the treatment for CHB, which may suppress viral replication, reverse liver fibrosis, attenuate the progression of liver disease, and reduce the risk of HCC.^4–6^ However, NUCs cannot effectively eradicate covalently closed circular DNA (cccDNA) in HBV-infected hepatocytes; hence, viral replication may recur after cessation of NUCs treatment.^7,8^

The ideal treatment endpoint for CHB is HBsAg seroclearance,^9,10^ but it could only be achieved in minority of patients, and long-term NUCs treatment is usually required.^11^ However, long-term NUCs treatment is limited by patient compliance, and safety of long-term treatment is of concern.^12^ Moreover, long-term NUCs treatment may result in a considerable financial burden on healthcare systems, and in many countries, patients are not fully reimbursed for the costs of NUCs treatment. In Taiwan, NUCs treatment is reimbursed by the National Health Insurance program for only 3 years in non-cirrhotic CHB patients, irrespective of the treatment response. Therefore, treatment cessation remains an inevitable issue in real-world practice.

In this regard, several studies have assessed the durability of treatment response and possibility for cessation of NUCs.^13–19^ For HBeAg-positive patients, it is generally accepted that NUCs treatment may be discontinued in patients with HBeAg seroconversion and have completed 6 to 12 months of consolidation therapy.^9,10,20^ For HBeAg-negative patients, although recent study reported that the virological relapse rate is high after cessation of NUCs,^16^ the study by Jeng et al^15^ demonstrated that the clinical relapse rate after 1 year of stopping entecavir treatment was 45.3%, suggesting that about half of the cases could stop NUCs treatment safely. Baseline serum HBV viral loads, HBsAg levels, and duration consolidation treatment have been shown to correlate with the risk of relapse in these patients.^13–15,18,19^ These data imply that cessation of NUCs might be possible in certain cases.

Whether discontinuation of NUCs is safe in selected CHB patients remains debated, and it is worth to identify those with lowest risk of relapse after treatment cessation. The aim of this study was to assess the durability of NUCs and predictors of sustained response after cessation of NUCs by National Health Insurance program in Taiwan.

## MATERIALS AND METHODS

### Patients

**FIGURE 1:**
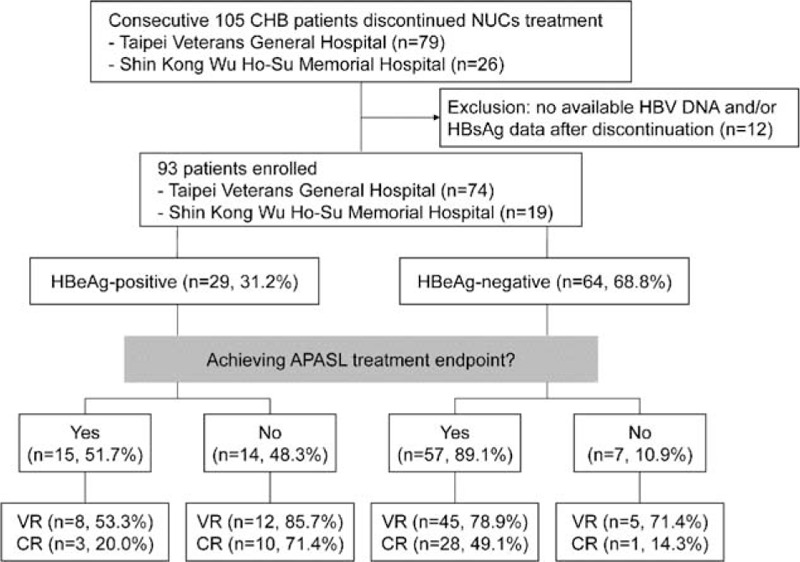
Patient selection and outcomes.CR = clinical relapse, VR = virological relapse.

From February 2012 to April 2014, consecutive 105 noncirrhotic CHB patients who discontinued NUCs treatment at 2 medical centers in Taiwan (Taipei Veterans General Hospital and Shin Kong Wu Ho-Su Memorial Hospital, Taipei, Taiwan) under the national health insurance program were retrospectively reviewed. Among them, 12 patients were excluded due to no available baseline HBV DNA and/or HBsAg data after discontinuation (Figure 1). All patients were positive for serum HBsAg for >6 months prior to NUCs therapy and fulfilled the treatment criteria for CHB according to the APASL treatment guidelines, that is, serum alanine aminotransferase (ALT) levels >80 U/L (2× upper limit of normal [ULN]) with HBV DNA >20,000 IU/mL for HBeAg-positive patients or >2000 IU/mL for HBeAg-negative patients.^20^ Patients with radiological evidence of cirrhosis or HCC were excluded. In general, the NUCs treatment was reimbursed for 3 years in both noncirrhotic HBeAg-positive and HBeAg-negative patients, which is under the regulation of National Health Insurance Administration, Ministry of Health and Welfare, Taiwan. In HBeAg-positive patients, additional 1 year of NUCs consolidation treatment was also reimbursed in patients achieving HBeAg seroconversion within 3 years of NUCs treatment. During a mean 52 weeks of follow-up, 255 times of HBsAg and HBV DNA measurements had been performed for the 93 patients. The mean interval of HBV DNA and HBsAg monitoring was 16.7 ± 7.7 weeks.

This study was approved by the Institutional Review Board, Taipei Veterans General Hospital, and Shin Kong Wu Ho-Su Memorial Hospital, which complied with standards of the Declaration of Helsinki and current ethical guidelines.

### Definition

The 2012 APASL treatment endpoint for NUCs was defined as HBeAg seroconversion with undetectable HBV DNA for at least 12 months in HBeAg-positive patients, and in HBeAg-negative patients treated for at least 2 years with undetectable HBV DNA documented on 3 separate occasions 6 months apart.^20^ Virological relapse (VR) was defined as an HBV DNA >2000 IU/mL, whereas clinical relapse (CR) was defined as HBVDNA >2000 IU/mL plus ALT elevation >2× ULN.^20^ Spontaneous remission was defined as spontaneous decline in HBV DNA to <2000 IU/mL in patients developing VR.

### Liver Biochemistry and Viral Serological Tests

Serum biochemical studies were performed using a systemic multiautoanalyzer (Technicon SMAC, Technicon Instruments Corp., Tarrytown, NY). The serum ALT ULN was set by the laboratory at 40 U/L for both males and females. The serum samples were tested for the presence of HBeAg and anti-HBe antibody using radio-immunoassay (Abott Laboratories, North Chicago, IL). HBV DNA was determined by Roche Cobas Tagman HBV DNA assay (detection limit of 12 IU/mL, Roche Diagnostics, Basel, Switzerland), whereas HBsAg levels were quantified using the Elecsys HBsAg II assay (detection limit of 0.05 IU/mL, Roche Diagnostics GmbH, Mannheim, Germany).

### Statistical Analyses

Values were expressed as median (ranges) or mean ± standard deviation when appropriate. Mann–Whitney *U* test was used to compare continuous variables. Pearson *χ*^2^ analysis or Fisher exact test was used to compare categorical variables. Cumulative HBV relapse rates were estimated by the Kaplan–Meier method and compared by the log-rank test. Analysis of predictive factors for relapse was performed using the Cox proportional hazards model. Variables with statistical significance (*P* < 0.05) or those close to significance (*P* < 0.1) by univariate analysis were subsequently included in the multivariate analysis. All statistical analyses were performed using the Statistical Package for Social Sciences (SPSS 17.0 for Windows, SPSS Inc, Chicago, IL). A 2-tailed *P* value <0.05 was considered statistically significant.

## RESULTS

### Patient Characteristics at the End of NUCs Treatment

Characteristics of the CHB patients at the end of NUCs treatment were summarized in Table 1. Among the 93 enrolled patients, 29 (31.2%) and 64 (68.8%) were HBeAg-positive and HBeAg-negative, respectively. Majority (65.5% in HBeAg-positive and 82.9% in HBeAg-negative) of patients received entecavir treatment. The median duration of NUCs treatment was 157 weeks.

**TABLE 1:**
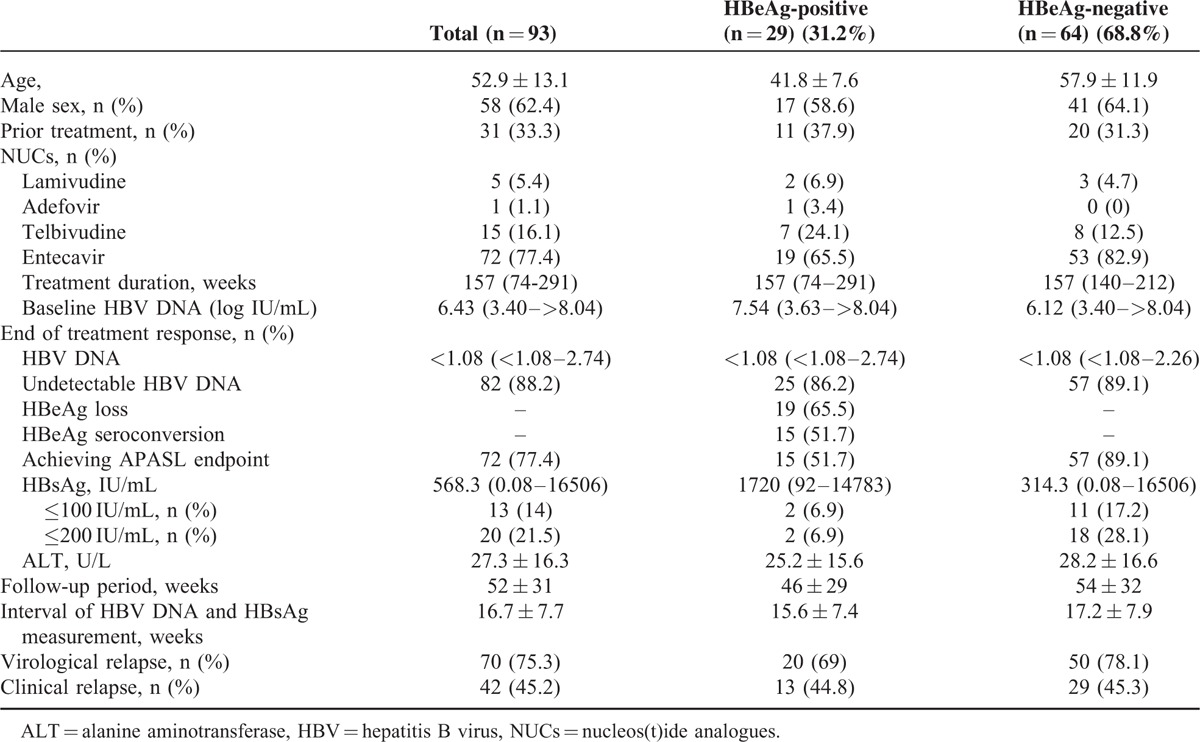
Characteristics of the CHB Patients

Overall, 51.7% of the HBeAg-positive and 89.1% of the HBeAg-negative patients achieved 2012 APASL treatment endpoints after a median 3 years’ treatment. As shown in Table 2, the baseline characteristics were comparable between HBeAg-positive patients with and without achieving APASL endpoint, whereas a higher proportion of the HBeAg-negative patients who achieved APASL treatment endpoint were treated with entecavir (*P* = 0.014).

**TABLE 2:**
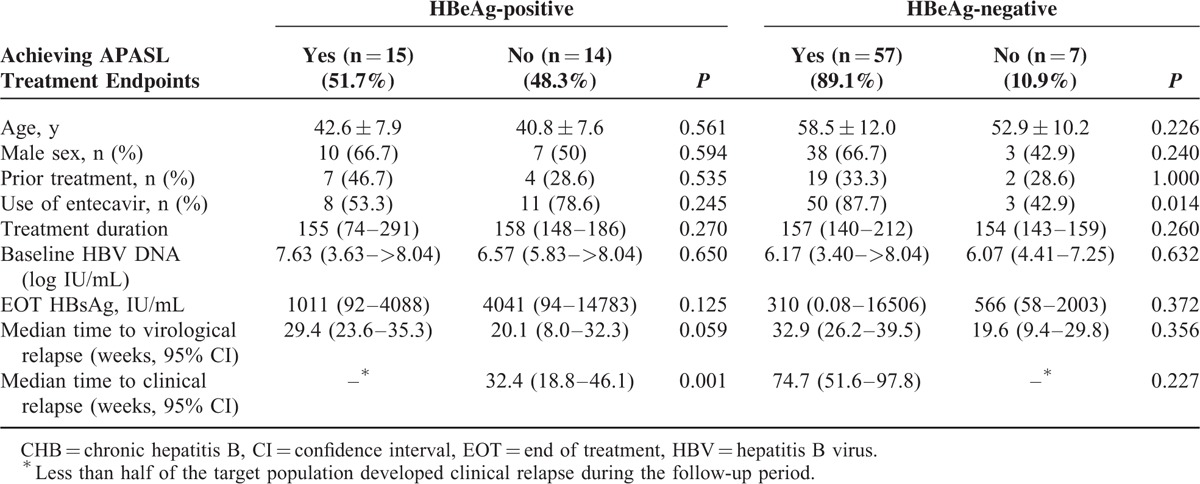
Characteristics of the CHB Patients With and Without Achieving APASL Treatment Endpoints

### Virological and Clinical Relapse Rates in Patients Achieving APASL Endpoints

**FIGURE 2:**
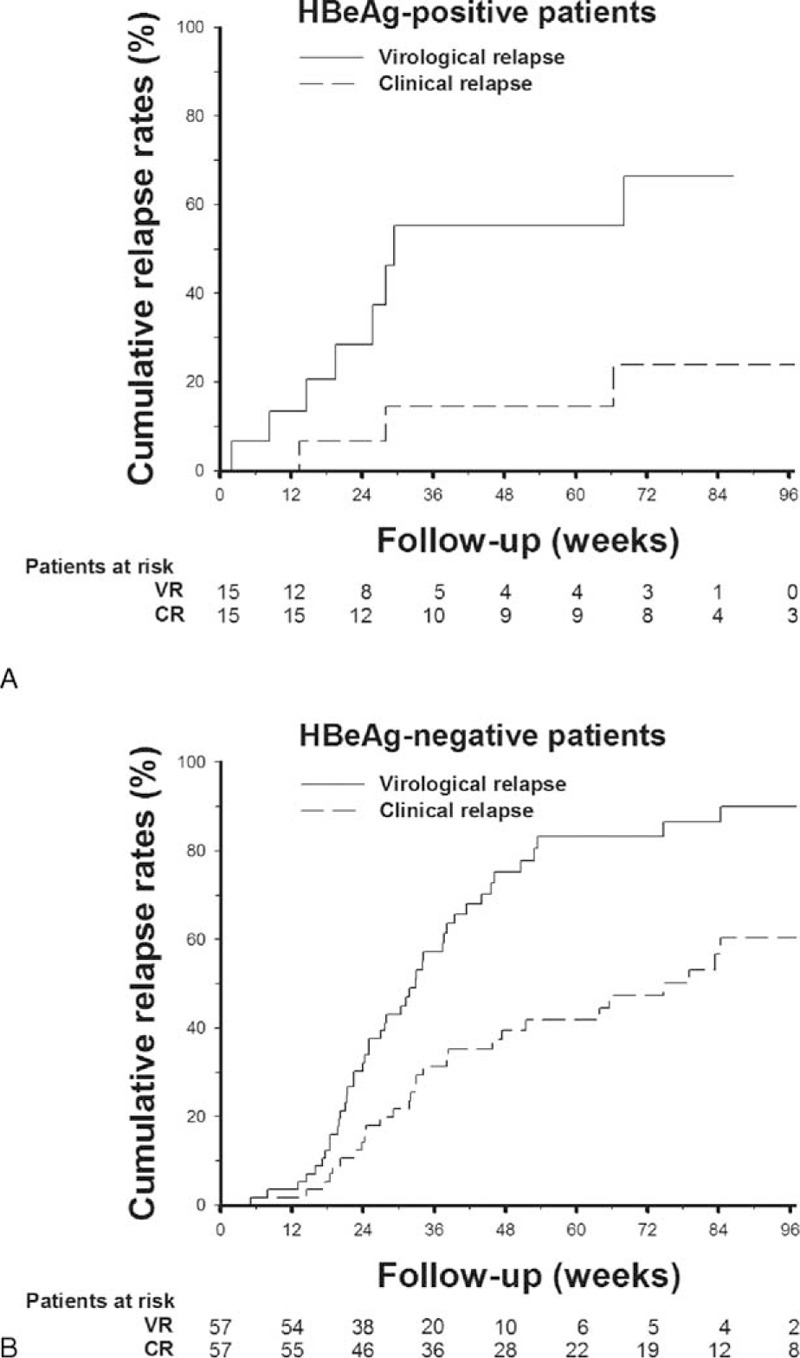
Cumulative rates of VR and CR after cessation of nucleos(t)ide analogues in patients with chronic hepatitis B and achieving APASL treatment endpoint. (A) VR and CR rates in HBeAg-positive patients. (B) VR and CR rates in HBeAg-negative patients. CR = clinical relapse, VR = virological relapse.

In 29 HBeAg-positive patients, VR and CR developed in 20 (69%) and 13 (44.8%) patients during the mean follow-up period of 46 weeks (Figure 1). HBeAg-positive patients achieving APASL treatment endpoint had a trend of longer median time to VR, and a significantly longer median time to CR, as compared with those without achieving APASL treatment endpoint (Table 2). In the 15 HBeAg-positive patients who achieved APASL treatment endpoint, the cumulative 1-year VR and CR rates were 55.3% and 14.4%, respectively (Figure 2A).

In the 64 HBeAg-negative patients, VR and CR developed in 50 (78.1%) and 29 (45.3%) patients, respectively, during the mean follow-up period of 54 weeks (Figure 1). The VR and CR rates were not statistically different between HBeAg-negative patients with and without achieving APASL treatment endpoint (Table 2). In the 57 HBeAg-negative patients who achieved APASL treatment endpoint, the cumulative 1-year VR and CR rates were 77.7% and 41.9%, respectively (Figure 2B).

### Predictors of Virological and Clinical Relapses in Patients Achieving APASL Treatment Endpoints

In the 15 HBeAg-positive patients who achieved APASL treatment endpoint with 12 months of consolidation treatment, there was no significant predictor of either VR or CR (Table 3). Baseline HBV viral loads, HBsAg levels at EOT, and potency of NUCs were not associated with the risk of HBV relapse.

**TABLE 3:**
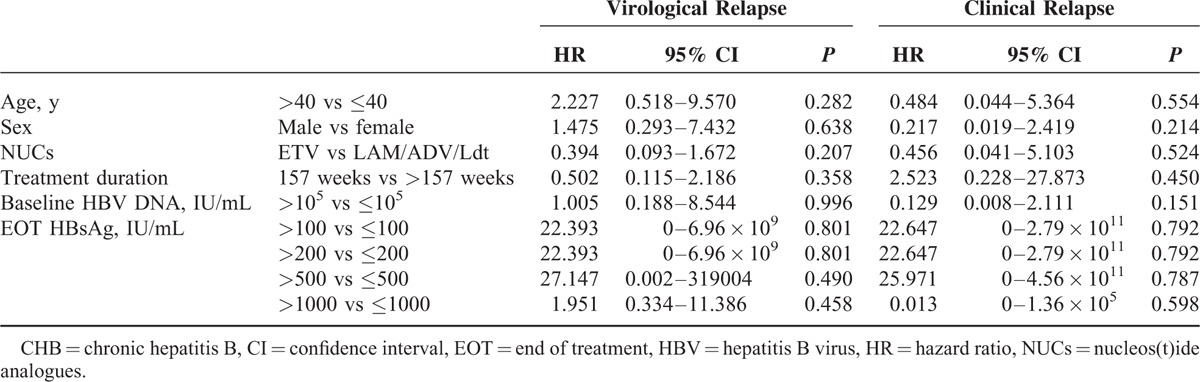
Univariate Analyses of Factors Associated With Virological and Clinical Relapses in 15 HBeAg-positive CHB Patients Achieving APASL Treatment Endpoint

**FIGURE 3:**
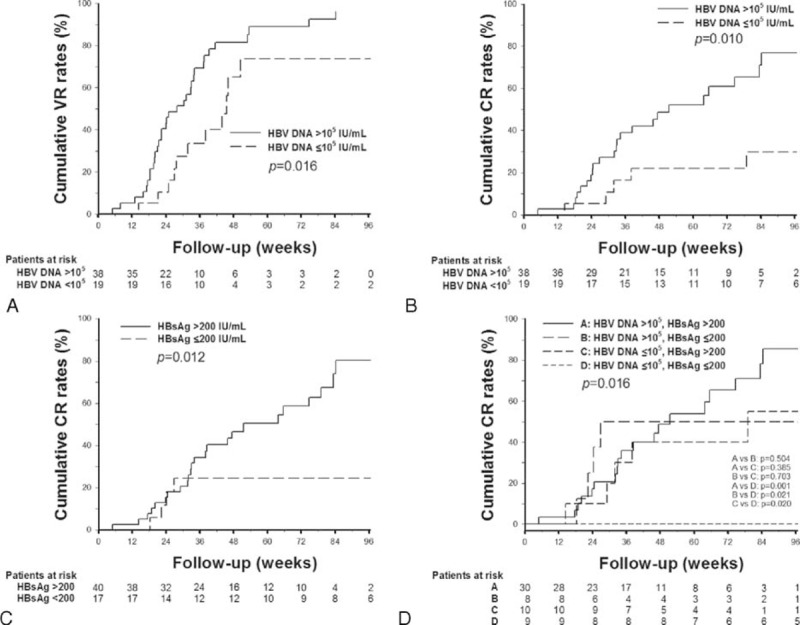
Cumulative rates of VR and CR in HBeAg-negative patients achieving APASL treatment endpoints. (A) Cumulative VR rates stratified by baseline viral loads. (B) Cumulative CR rates stratified by baseline viral loads. (C) Cumulative CR rates stratified by end of treatment HBsAg levels. (D) Cumulative CR rates stratified by baseline viral loads and end of treatment HBsAg levels. CR = clinical relapse, VR = virological relapse.

In the 57 HBeAg-negative patients who achieved APASL treatment endpoint, baseline HBV DNA >10^5^ IU/mL was the only predictor of VR (hazard ratio [HR] = 2.277, *P* = 0.019, Table 4). The cumulative 1-year VR rates were as high as 81.5% and 73.8%, respectively, in patients with baseline HBV DNA higher and lower than 10^5^ IU/mL (*P* = 0.016, Figure 3A). For the risk of CR, baseline HBV DNA >10^5^ IU/mL (HR = 3.378, *P* = 0.014, Figure 3B) and HBsAg levels >200 IU/mL at EOT (HR = 3.661, *P* = 0.018, Figure 3C) were factors significantly associated with CR in univariate analysis (Table 4). In multivariate analysis, baseline HBV DNA >10^5^ IU/mL was the only independent predictor of CR.

**TABLE 4:**
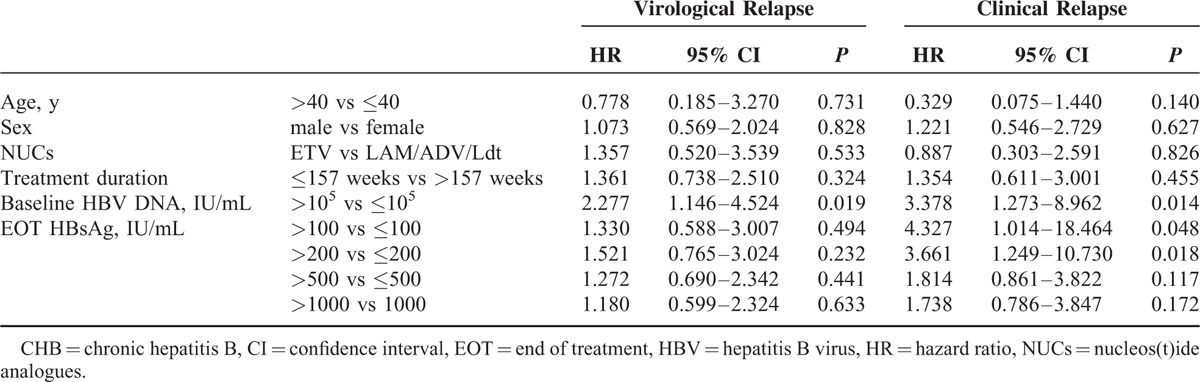
Univariate Analyses of Factors Associated With Virological and Clinical Relapses in 57 HBeAg-negative CHB Patients Achieving APASL Treatment Endpoint

### Outcomes of Patients Who Developed Virological Relapse

**FIGURE 4:**
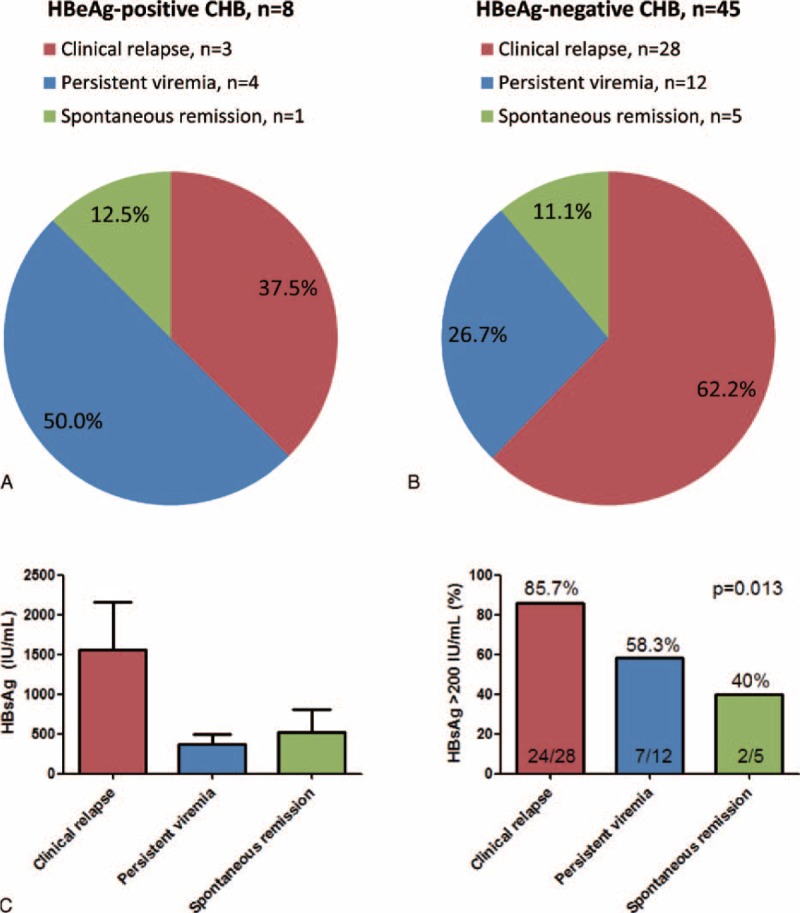
Outcomes of patients who achieved APASL treatment endpoints but developed virological relapse (VR). (A) Outcomes in HBeAg-positive patients with VR. (B) Outcomes in HBeAg-negative patients with VR. (C) Left panel: Mean HBsAg levels in each group of HBeAg-negative patients with VR. Right panel: Proportion of patient with HBsAg >200 IU/mL in each group of HBeAg-negative patients with VR. Persistent viremia was defined as persistent HBV DNA >2000 IU/mL without clinical relapse. Spontaneous remission was defined as spontaneous decline of HBV DNA to less than 2000 IU/mL in patients developing VR.

Eight of the 15 HBeAg-positive patients who achieved APASL treatment endpoint developed VR. Among them, 3 (37.5%) subsequently developed CR, 4 (50%) remained viremic with HBV DNA >2000 IU/mL without developing CR, and 1 (12.5%) had spontaneous remission with the HBV viral load <2000 IU/mL (Figure 4A). Forty-five of the 57 HBeAg-positive patients who achieved APASL treatment endpoint developed VR. Among them, 28 (62.2%) subsequently developed CR, 12 (26.7%) remained viremic with HBV DNA >2000 IU/mL without developing CR, and 5 (11.1%) had spontaneous remission to inactive HBV carrier status (Figure 4B). The mean HBsAg levels at EOT among the HBeAg-negative patients with subsequent CR, persistent viremia, and spontaneous remission were 1560, 376, and 519 IU/mL, respectively (Figure 4C, left panel), whereas the proportion of HBsAg >200 IU/mL were 85.7%, 58.3%, and 40% among the 3 outcomes of patients, respectively (*P* = 0.013, Figure 4C, right panel).

### Selection of Patients With Lower Risk of Clinical Relapse

Of the 45 HBeAg-negative patients who developed VR, HBsAg >200 IU/mL at EOT was also the independent predictor of CR (HR = 3.573, 95% confidence interval = 1.190–10.733, *P* = 0.023). Importantly, patients with baseline HBV DNA ≤10^5^ IU/mL and EOT HBsAg levels ≤200 IU/mL had a significantly lower risk of CR (*P* = 0.016, Figure 3D). All the 9 patients with baseline HBV DNA ≤10^5^ IU/mL and EOT HBsAg levels ≤200 IU/mL did not develop CR during the follow-up period.

## DISCUSSION

In this study, we observed a high rate of VR after cessation of NUCs treatment in both HBeAg-positive and -negative CHB patients, and the risk of CR remained high in HBeAg-negative patients who achieved APASL treatment endpoints.

Patients who stopped NUCs after HBeAg seroconversion with 12 months’ consolidation therapy had 1-year VR and CR rates of 55.3% and 14.4%, respectively. The 1-year VR rate was comparable with that in previous studies.^13,19^ In the study by Dai et al^14^, about 60% of the HBeAg-positive patients who stopped lamivudine after achieving the APASL treatment endpoint could not maintain combined HBeAg-seroconversion and HBV DNA undetectable at 6 months after EOT. Similar to this finding, in our study, 10 of the 15 HBeAg-positive patients (66.7%) who achieved APASL endpoint failed to maintain combined virological response at 6 months after EOT, including 1 case with HBeAg reversion. More than half of the HBeAg-positive patients developed VR despite achieving APASL endpoint, but only minority of them developed CR within 1 year. Therefore, these findings suggested that the current APASL stopping rule for HBeAg-positive patients, which is also consistent with the current AASLD and EASL treatment guidelines, is clinically feasible based on low risk of CR in short term. Consolidation treatment is an important predictor of HBV relapse in HBeAg-positive patients. Dai et al^14^ recently showed that longer duration of consolidation therapy, lower pretreatment viral loads, and HBsAg levels were significantly associated with combined response at 6 months after cessation of lamivudine in HBeAg-positive patients. Another recent study by Chen et al^19^ showed that only age was a predictor of VR after cessation of lamivudine treatment. In our study, due to the limitation of case number, we did not identify a significant predictor of VR in HBeAg-positive patients. Nevertheless, considering the high VR rate in HBeAg-positive patients and the risk of disease progression in those with persistent viremia, longer consolidation treatment to reduce the risk of VR should be considered in HBeAg-positive patients.

Only minority of the HBeAg-negative patients failed to achieve APASL treatment endpoint after 3 years of NUCs treatment. A recent study by Seto et al^16^ also reported a 1-year VR rate of 91.4% after cessation of entecavir treatment in HBeAg-negative patients. Therefore, the stopping rule suggested by the 2012 APASL guidelines has potential high-risk of VR for HBeAg-negative patients. To avoid the risk of VR, an indefinite duration of NUCs for these patients until HBsAg seroclearance should be considered, as recommended by the current AASLD and EASL treatment guidelines.^9,10^ Our 1-year CR rate of 41.9% in HBeAg-negative patients achieving APASL treatment endpoint, with nearly 83% of them receiving entecavir treatment, was comparable with previous reports.^15,17^ In the study by Jeng et al,^15^ the 1-year CR rate was 45% after entecavir treatment, and they suggested that with proper off-therapy monitoring, the APASL stopping rule for HBeAg-negative CHB patients is generally safe. However, the study did not emphasize the VR rates, which were high in our observation and previous report,^16^ and the outcomes for these patients with persistent viremia were still unclear. In consideration of the risk of disease progression and HCC in patients with high HBV viral load,^21,22^ finite NUCs treatment is debated.

The predictors of HBV relapse in HBeAg-negative patients were controversial. The study by Chen et al^19^ showed that HBsAg levels at EOT were independently associated with VR after cessation of lamivudine in HBeAg-negative patients. However, in the prospective study by Seto et al^16^, various HBsAg cutoff levels at EOT could not predict VR after cessation of entecavir. In our study, we identified baseline HBV viral loads as the only predictor of VR in HBeAg-negative patients. Consistent with the study by Jeng et al^15^, baseline HBV viral load was the only independent predictor of CR. In our study, HBsAg level at EOT was the only predictor of CR after developing VR. Previous studies suggest that lower serum HBsAg levels at EOT might reflect a better host immunological control of HBV,^11,23^ and therefore the risk of subsequent hepatitis flare could be lower. We further stratified the patients according to their baseline viral loads and EOT HBsAg levels, and showed that patients with baseline HBV DNA <10^5^ IU/mL and EOT HBsAg levels <200 IU/mL had a lowest risk of CR (Figure 3D). None of the 9 patients who fulfilled this criterion developed CR during the follow-up period.

NUCs with higher antiviral potency were suggested to have a better durability of treatment response. In this study, 77% of the patients received entecavir treatment, and the case number for those receiving lamivudine, adefovir, or telbivudine was smaller. Therefore, the issue was not further analyzed in this study.

The outcomes of patients who developed VR despite achieving APASL treatment endpoints were rarely reported. In this study, about 11% to 12% of patients have a chance of spontaneous remission after developing VR, defined as spontaneous decline of HBV DNA to <2000 IU/mL in both HBeAg-negative and -positive patients. Due to small case number, there were no significant factors predicting the outcome after VR in HBeAg-positive patients. In HBeAg-negative patients, we identified HBsAg >200 IU/mL at EOT as the predictor of subsequent CR (Figure 4C) and low HBsAg at EOT was found in patients with subsequent remission, which could serve as an additional biomarker to select patients with the risk of CR.

This study has some limitations. First, the case number is small despite recruiting patients from 2 medical centers in Taiwan. However, patients who did not achieve APASL treatment endpoints might decide to continue NUCs treatment, whereas most patients received indefinite NUCs until HBsAg seroclearance, according to current AASLD and EASL guidelines. Second, this is a retrospective study. However, due to the alert of high relapse rates, most patients were closely monitored with HBV viral loads and HBsAg levels at a mean interval of 4 months after EOT in this study, which could be avoid of the possibility of underestimation for the relapse rate. Third, NUCs with higher antiviral potency were suggested to have a better durability of treatment response. In this study, 77% of the patients received entecavir treatment, and the case number for those receiving lamivudine, adefovir, or telbivudine was smaller. Therefore, the issue was not further analyzed in this study.

In conclusion, the risk of VR is high after cessation of NUCs treatment despite achieving APASL treatment endpoint in both HBeAg-positive and -negative CHB patients. Current APASL treatment endpoint is feasible for HBeAg-positive patients based on low risk of CR, but careful off-treatment monitoring is still needed. Cessation of NUCs therapy could only be considered only in selected HBeAg-negative patients with low baseline viral loads and low EOT HBsAg levels after achieving APASL treatment endpoint.
